# MS treatment de-escalation: review and commentary

**DOI:** 10.1007/s00415-024-12584-x

**Published:** 2024-08-02

**Authors:** Krzysztof Selmaj, Hans-Peter Hartung, Marcin P. Mycko, Igor Selmaj, Anne H. Cross

**Affiliations:** 1 Department of Neurology, University of Warmia & Mazury, Olsztyn, Poland; 2 Center of Neurology, Lodz, Poland; 3 Department of Neurology, Heinrich-Heine-University, Düsseldorf, Germany; 4 Department of Neurology, Medical University of Vienna, Vienna, Austria; 5 Department of Neurology, Palacky University, Olomouc, Czech Republic; 6 Brain and Mind Center, University of Sydney, Sydney, Australia; 7 Washington University School of Medicine, St. Louis, MO USA

**Keywords:** Multiple sclerosis, Disease-modifying therapies, De-escalation, Immunosenescence

## Abstract

Almost all currently licensed disease-modifying therapies (DMTs) for MS treatment require prolonged if not lifelong administration. Yet, as people age, the immune system has increasingly reduced responsiveness, known as immunosenescence. Many MS DMTs reduce the responsiveness of the immune system, increasing the risks for infections and possibly cancers. As people with MS (pwMS) age, it is recognized that inflammatory MS activity declines. Several studies have addressed de-escalation of DMTs for relapsing MS under special circumstances. Here, we review evidence for de-escalating DMTs as a strategy that is particularly relevant to pwMS of older age. Treatment de-escalation can involve various strategies, such as extended or reduced dosing, switching from high-efficacy DMTs having higher risks to moderately effective DMTs with lesser risks, or treatment discontinuation. Studies have suggested that for natalizumab extended dosing maintained clinical efficacy while reducing the risk of PML. Extended interval dosing of ocrelizumab mitigated the decline of Ig levels. Retrospective and observational discontinuation studies demonstrate that age is an essential modifier of drug efficacy. Discontinuation of MS treatment in older patients has been associated with a stable disease course, while younger patients who discontinued treatment were more likely to experience new clinical activity. A recently completed 2-year randomized-controlled discontinuation study in 260 stable pwMS > 55 years found stable clinical multiple sclerosis with only a small increased risk of new MRI activity upon discontinuation. DMT de-escalation or discontinuation in MS patients older than 55 years may be non-inferior to continued treatment with immunosuppressive agents having higher health risks. However, despite several small studies, a definite conclusion about treatment de-escalation in older pwMS will require larger and longer studies. Ideally, comparison of de-escalation versus continuation versus discontinuation of DMTs should be done by prospective randomized-controlled trials enrolling sufficient numbers of subjects to allow comparisons for MS patients of both sexes within age groups, such as 55–59, 60–65, 66–69, etc. Optimally, such studies should be 3 years or longer and should incorporate testing for specific markers of immunosenescence (such as T-cell receptor excision circles) to account for differential aging of individuals.

## Introduction

Multiple sclerosis (MS) is a chronic, progressive disorder of the CNS with a complex pathology that involves inflammation, demyelination, and axon injury. Several disease-modifying treatments (DMTs) for MS have been introduced in recent years [1]. These drugs primarily reduce relapse rates and new or gadolinium-enhancing CNS lesions on MRIs. Despite different mechanisms of action, almost all current MS DMTs have been often used for prolonged if not lifelong administration. Long and continuous treatments of people with MS (pwMS) with drugs having side effects or affecting the immune system may not be needed and could be detrimental. Thus, a new question has arisen: Should MS treatment at some point be de-escalated or discontinued? These speculations were supported by open-label extension and real-world studies showing that pwMS treated with DMTs of high efficacy had very low annual relapse rate (ARR) and limited progression of disability for several years after study completion [2, 3]. This observation is particularly relevant to older MS patients who are characterized by lower relapse rate and diminished response to treatments and higher risk of treatment-related adverse outcomes like infections and malignancies [4]. At the same time, there is lack of data about safety and efficacy in almost all randomized clinical trials (RCT) for patients with age over 55 years. In this paper, we present available data on DMT de-escalation and present the view that for some treated pwMS without disease activity or disability worsening, de-escalation might be considered. We reemphasize the need for assessing prognostic factors, MRI, and close monitoring following treatment de-escalation or cessation.

## Immunosenescence in MS

It is well documented that new neuro-inflammatory MS lesions decline with age. This is thought to be due, at least in part, to changes in the aging immune system. Immunosenescence was originally defined as remodeling of immune system structure occurring in both the adaptive and innate immune systems. Immune system dysfunction with aging leads to increased susceptibility to infection, reduced responses to vaccinations, increased rates of age-related diseases including neurodegenerative disorders, some autoimmune diseases, and malignancies [5]. In older pwMS, MS clinical [6] and MRI activity [7] diminish. In a study of 2477 patients, the relapse rate decreased by 17% every 5 years, between years 5 and 30 post-MS onset, and this decline increased in magnitude with increasing onset age [8]. A retrospective cohort study found that patients with renewed MS disease activity following DMT discontinuation were younger (median 53.6 years) than those who remained activity free [9].

In an older MS population, several characteristics of immunosenescence might have impact on MS course and responses to treatments (Table 1). With aging, naïve T and B cells decline, with associated declines in the diversity of TCR and BCR repertoires [10]. The decline in diversity of T cells begins around age 40 years; by age 80, the naïve T-cell repertoire may decline as much as 50% of what it was at the younger age. Aged CD4 + T cells express less CD40L, which leads to diminished humoral responses. Despite the continued presence of memory T and B cells, these changes make older people more vulnerable to infections [11], including severe infections [12, 13] and decreased vaccination responsiveness [14]. Unsurprisingly, the increased risk of infections was observed with some DMTs with highest risk for rituximab, fingolimod, and natalizumab [15, 16]. The increased risk of infections was intensified with increased disability, lymphopenia, hypogammaglobulinemia, and treatment duration [17]. Age was not found as an unequivocal cofactor for higher rate of infections during DMT [18]. Opportunistic infections, including PML, are also more frequent in older pwMS [19].

**Table 1:** Immunosenescence and MS

**Immune and metabolic alternations with immunosenescence:**
Reduction in the number of naive T cells
Accumulation of memory T and B cells
Chronic microglia activation
Mitochondrial dysfunction
Oxidative stress
DNA damage
Shortened leukocyte telomere length
**General clinical consequences of immunosenescence:**
Increased rate of infections, including increased risk of opportunistic infections
Decreased responsiveness to vaccination
Increased comorbidity burden
Increased rate of neoplasms
**MS-specific clinical consequences of immunosenescence:**
Decreased number of relapses
Increased disability progression
Diminished effectiveness of DMTs
Higher number of chronic active MRI lesions

Another aspect of immunosenescence is an increased risk of malignancy in aging MS patients. Meta-analyses of 45 randomized DMT trials revealed age as a contributor to higher incidence of neoplasms in pwMS treated with some cell depleting drugs especially above age 45 years [18]. However, the US Food and Drug Administration Adverse Event Reporting System database detected no definite safety signal for increased cancer risk among the approved DMTs for the MS population [20]. In addition, in a Swedish nationwide register-based cohort study, no increased risk of cancer was seen with rituximab and natalizumab, but a borderline-significant increased risk was observed with fingolimod, compared to the general population [21]. A long-term, continuous treatment with ocrelizumab for several years demonstrated no increased risk of malignancy (including female breast cancer) compared to matched controls from a the general population [22]. Similarity, long-term safety data from the cladribine tablets’ clinical development program did not confirm higher risk for cancer [23].

Another important phenomenon related to immunosenescence is its potential impact on effectiveness of DMTs in older patients(Table 2). A meta-analysis of 38 randomized, blinded MS clinical trials revealed that efficacy of DMTs on MS disability progression strongly decreased with advancing age. The regression model predicted no efficacy beyond mean age 53 years. Further, high-efficacy drugs outperformed low-efficacy drugs in reducing MS disability only for patients younger than 40.5 years [24]. A meta-analysis of six randomized-controlled trials with RRMS that reported subgroup analysis showed that patients in the < 40 age group achieved significantly higher reduction of disease activity than those in the ≥40 age group [25]. With the analyses from a retrospective, real-world cohort of MS patients to model disease activity for oral (dimethyl fumarate and fingolimod) and higher efficacy infusible (natalizumab and rituximab) DMTs by age, those patients ≥ 45 years of age on oral DMTs had no significant difference in disease activity compared to patients on infusible DMTs [26]. Thus, age is a modifier of DMT efficacy.

**Table 2:** Diminished effectiveness of DMT with age

Study citation	Study type	DMT	Outcome	Efficacy cut of age
Weideman et al. [24]	Meta-analysis of randomized, blinded 38 DMT trials	13 categories of DMT	Strong correlation between IDP and age	53 years; HET outperformed LET < 40.5 years
Signori et al. [25]	Meta-analysis of 6 randomized controlled DMT trials	NTZ, IFNb, TERF, DMF	ARR and CDP reduction associated with younger age	RE < 40 and > 40 years 0.83 vs 1.30 for ARR and 0.82 vs 1.28 CDP
Vollmer et al. [26]	Single-center retrospective real-word cohort	DMF,Fingo vs NTZ, RTX	Higher OR (= 2.82) for relapses on oral DMT < 45 yrs	HET overperformed LET only < 54.2 years

*IDP* inhibition of disability progression; *RE* relative effect; *HET* high-efficacy treatment; *LET* low-efficacy treatment; *ARR* annual relapse rate; *CDP* confirmed disability progression; *OR* odds ratio; *IFNb* beta interferon; *TERF* teriflunomide; *DMF* dimethyl fumarate; *Fingo* fingolimod; *NTZ* natalizumab; *RTX* rituximab

Aging of the MS population is even more significant because of increasing longevity within recent decades [27]. The mean age of the British Columbia MS cohort increased by 10 years between 1992 and 2008 [28]. A study using 2010 data estimated 29% of the U.S. MS population to be older than 65 years [29].

## De-escalating from a drug of high efficacy to a less-risky drug with lower efficacy

One suggested strategy for treating newly diagnosed people with MS, especially those with a concerning prognostic profile, is to initiate high-efficacy treatment early [30]. Randomized prospective studies are underway to better address this question (DELIVER-MS NCT03535298, TREAT-MS NCT03500328). However, the high-efficacy DMTs usually have more risks. These “high efficacy” DMTs for MS are: mitoxantrone, alemtuzumab, natalizumab, ocrelizumab, ofatumumab, and ublituximab. Many experts also include oral cladribine and sphingosine-1-phosphate receptor (S1PR) modulators in the high-efficacy category.

Early studies of this type compared outcomes following mitoxantrone for 6 months and then switched to interferon-beta (IFNβ)-1b, a safer DMT of lower efficacy versus treatment with IFNβ-1b for 3 years. The 3-year risk of worsening disability and relapse rate were reduced by 65% and 61,7% in the mitoxantrone→IFNβ-1b group by 65% and 61.7% respectively [31]. Overall, over 70% of patients switched to the IFNβ-1a remained relapse free over 96 weeks [32].

Natalizumab is a monoclonal antibody targeting α4β1 integrins, which is highly effective for relapsing MS, but carries risks including progressive multifocal leukoencephalopathy (PML), a life-threatening viral brain infection without an effective treatment. Several studies evaluated de-escalation therapy in which natalizumab treatment was stopped due to the risk of PML in pwMS with high JC virus (JCV) index. Stopping natalizumab without immediate initiation of another DMT often results in renewed or even excess relapse activity. In earlier small studies of de-escalating from natalizumab to IFNβ or glatiramer, there was an association with clinical and radiological disease recurrence [33, 34]. Switching to fingolimod, an S1PR modulator with moderate-to-high efficacy, prevented post-natalizumab relapse risk in 637 pwMS. Patients with ≤ 8 week washout period had a markedly lower risk of relapses, 4.5%, than those with > 8 weeks, 51.4% [35]. It should be emphasized that switching from natalizumab to fingolimod requires short or even no washout periods. However, comparison between anti-CD20 monoclonal antibodies and fingolimod showed that rituximab and ocrelizumab use was associated with the lowest ARR and discontinuation rates, the longest time to first relapse, and the lowest disability accumulation [36, 37]. Results thus far indicate that relapsing MS activity after natalizumab discontinuation is not reliably prevented with de-escalation to lower efficacy drugs.

## Extended interval dosing

Prolonged use of certain MS drugs incurs health risks. Prolonged use of natalizumab in JCV antibody positive patients increases PML risk. Although the best way to reduce PML risk is to use this drug in JCV seronegative patients and switched off natalizumab after seroconversion recent studies suggest that extended dosing might reduce the risk of PML. Pharmacodynamic studies suggested that α4β1 integrin saturation rate with standard natalizumab 300 mg every 4 week dosing might exceed the requirement to control mononuclear cell adhesion and clinical outcomes [38]. Both analytical modeling [39] and TOUCH registry data [40] suggested that lengthened intervals between natalizumab infusions reduced PML risk without mitigating efficacy. No differences in relapse rate and MRI activity were noted in a 2014 retrospective report [41] comparing 96 pwMS treated for 20 ± 11 months with natalizumab every 6–8 weeks versus 265 people who remained on every 4-week dosing. These results were confirmed in a large multi-center study [42]. Two large Italian studies also did not find differences in ARR, disability worsening, and number of active MRI lesions between pwMS treated with standard versus extended dosing [43, 44]. Data from TOP showed that 210 pwMS switched to every 6-week dosing did not have increased ARR and disability worsening compared to pwMS on regular natalizumab dosing [45]. Similarly, data from the MS PATH registry showed no differences in several MRI parameters including brain atrophy in pwMS treated with extended administration intervals versus every 4 weeks [46]. The NOVA study was the first randomized, controlled, phase 3b trial evaluating the efficacy of natalizumab administrated once every 6 weeks [47]. This study found insignificant differences in numbers of new and enlarging T2 lesions between patients treated with extended versus standard dosing. Additionally, relapse rate, EDSS, timed 25-Foot Walk, 9-Hole Peg Test and Symbol Digit Modalities Test, and patient-reported outcomes confirmed the non-inferiority of every 6-week versus 4-week dosing [48]. Further, serum neurofilament-light chain levels were similar in pwMS undergoing 6- or 4-week interval dosing [49]. The risk of PML in pwMS receiving extended dosing of natalizumab was compared retrospectively to pwMS on standard dosing in the large cohort of TOUCH registry. The covariate-adjusted reduction in PML risk was 94% for the extended-interval group [40].

Ocrelizumab is a “high efficacy DMT” monoclonal antibody that lyses cells expressing CD20, leading to virtually no circulating B lymphocytes. Ocrelizumab treatment is associated with increased infection risk, including a higher risk of severe COVID-19. During the peak of the COVID-19 pandemic, several MS centers delayed ocrelizumab drug infusions, aiming to reduce the impact of ocrelizumab on the immune system. In a German retrospective, multi-center cohort study, 116 patients received ocrelizumab on extended-interval regimen (median delay 8.68 weeks), and 90.5% patients with extended dosing remained relapse free. Progression of disability was observed in 8.9% on standard and 9.5% on a delayed regimen. MRI progression was documented in 4.5% and 6.9%, respectively [50]. A retrospective clinical audit in one UK site found no significant differences in relapse rate or radiological activity among 67 patients who received extended-interval dosing (≥ 30 weeks) compared to standard interval dosing (< 30 weeks) [51]. An Italian study reported data on 213 RRMS and 61 PPMS patients treated according to the standard ocrelizumab regimen or with > 4 week delay. In pwRRMS, a trend but no statistically significant worsening was found in clinical relapses (9.6% vs 16.3%), MRI activity (9.8% vs 14.1%), and disability progression (11.3% vs 18.4%), respectively. Similar findings were observed in pwPPMS [52]. In another study assessing dosing of ocrelizumab in pwRRMS and pwPMS with extended time from last dose to 8 or 12 months, no PMS patients had breakthrough disease activity. Among the RRMS groups, equal low number of patients had new disease activity [53]. All these data are in accord with the results of the Ocrelizumab phase 2 extension study in which no new enhancing MRI lesion and no increased risk of relapse parallel to substantial drop in infections were observed 18 months after drug discontinuation [54].

One of the major adverse effects of ocrelizumab and other anti-CD20 treatments is hypogammaglobulinemia of IgM and IgG. A pilot study [55] found that adapting the dosing interval to B-cell depletion (“B-cell-adapted”) such that doses were delayed until B cells were > 1% of blood lymphocytes extended the mean time until next ocrelizumab infusion from 27.3 to 46.1 weeks. Over 12 months, Ig levels declined significantly in the standard interval dosing group but not in the “B-cell adapted” dosing group and without seeming to affect disease activity. In another study, extended dosing interval did not lead to increased repopulation of effector B cells, memory B cells, or plasmablasts, but higher percentages of transitional, naïve, and regulatory B cells were observed [56]. Importantly, changes in B-cell subsets after both dosing schemes did not correlate with MS relapses or progression. In another study, B-cell repopulation-guided interval dosing [57] extended the average infusion intervals to 319 days and found no significant difference in No Evidence of Disease Activity-3 (NEDA-3), 90.4% vs 83.3%, respectively, or in hypogammaglobulinemia IgG rates versus standard dosing. IgM hypogammaglobulinemia was significantly less common in the extended (17.3%) vs standard (55%) group. It was concluded that ocrelizumab dosing intervals guided by B-cell repopulation is a safe alternative to the traditional dosing with similar efficacy and might significantly lower hypogammaglobulinemia rates.

Similarly, in a prospective observational study, 718 pwRRMS were evaluated after implementation of extended rituximab dosing. Difference in hazard ratio for relapse when comparing dosing  ≥ 8 to 12,  ≥ 12 to 18, and  ≥ 18 months with < 8 months was non-significant. Neuroradiologic outcomes mirrored relapse rates [58].

Overall, these studies of extended dosing of anti-CD20 B-cell lytic monoclonal antibodies showed maintained clinical efficacy while concurrently improving IgM and IgG levels (Table 3). Two studies are underway to prospectively assess altered dosing of ocrelizumab: annual versus biannual infusions (WINDOCRE, NCT05999604), and B-cell tailored dosing (BLOOMS, NCT05296161).

**Table 3:** Studies on efficacy of extended dosing in MS treatment

Investigators, study citation	Type of study	Population, *N*, SID vs EID	Extended interval time	Clinical and lab outcomes SID vs EID	MRI outcome SID vs EID
**Natalizumab**					
Bomprezzi and Pawate [41]	Retrospective review from 2 MS centers	RRMS 265 vs 96	6–8 weeks	ARR 13 vs 13%	New activity lesion 11 vs 9%
Ryerson et al. [42]	Retrospective review from 9 MS centers	RRMS 1080 vs 894	3–61 days	ARR 0.14 vs 0.08 NEDA3 62% vs 61%	NET2L 17% vs 14% GdE 7% vs 9%
De Mercanti et al. [43]	Retrospective, multi-center cohort study, to 2 years or loss of follow-up	RRMS 187 vs 129	< 5 weeks SID group (MDI = 4.5 wks) and > 5 wks (MDI 6.1 wks)	*NA*	NET2L and GdE 8.5% vs 6.55%
Chisari et al. [44]	Retrospective multi-center, to 24 month follow-up	RRMS 1254 vs 838	Median 43 days	ARR 0.09 vs 0.1 EDSS > 2 6.5%vs 5.5% CDI 17.6% vs 14.9% NEDA2 79.3% vs 79.1%	NA
TOP [45]	Observational propensity score matched	RRMS 291 vs 291	6 weeks	ARR 0.157 vs 0.150 Relapse free HR = 1.24 CWD HR = 0.786	NA
MS PATH [46]	Multicenter standardized acquisition protocols	RRMS 299 vs 66	> 35 days	NA	NET2L OR = 1.07 NET2L 0.60 vs 0.96 Diff. BPF −0.11%
NOVA [47]	Randomized, multi-center, phase 3b, 72 weeks	RRMS 248 vs 251	6 weeks	Time to relapse HR = 1.31 CWD HR = 1.29 PML 0 vs 1 case	NET2L 0.05 vs 0.2
TOUCH [40]	Retrospective cohort study	RRMS 13,132 vs 1988	35–43 days	PML risk reduction 94% for EID	NA
**Ocrelizumab**					
German cohort [50]	Retrospective, five center cohort study with 3 month follow-up	RRMS 202 vs 116	MDI 8.69 month	Relapse free 90.1% vs 90.5% CWD 8.9% vs 9.5% B-cell depletion 82.2% vs 83.3%	NET2L/GdE 4.5% vs 6.9%
UK study [51]	Retrospective electronic records with 4 month follow-up	RRMS/PPMS 69 vs 67	Mean 48.3 weeks	Relapse rate 4.3% vs 3.0% B-cell > 1 17% vs 94% (*p* 0.001)	NET2L 8.7% vs 7.5% GdE 0 vs 4.3%
San Raffaele [52]	Retrospective cohort study with 1.28 year follow-up	RMS + PPMS 144 vs 130	> 4 weeks	Relapse rate in RMS 9.6% vs 16.3% CWD 11.3% vs 18.4%	Active 9.8% vs 14.1%
Schuckmann et al [55]	Single-center B-cell adapted study	RMS 14 vs 12	46.1 weeks	IgG -3.6% vs 0.1% IgM −11.5% vs + 5.5% IgA −6.4% vs −1.9% No difference in NEDA-3, EDSS, SDMT, NFL	NA
Rempe et al. [57]	Two-center retrospective B-cell adapted study	RRMS,PPMS, SPMS 60 vs 52	319 days	NEDA-3 83.3% vs 90.4% Hypo-IgG 6.7% vs 1.9% Hypo-IgM 55% vs 17.3%	NA
Rjeily et al. [53]	Retrospective cohort study	RRMS + PPMS 130 vs 273/88	> 8 months > 12 months	RRMS relapse rate 1.5% vs 0.3% PPMS 0 vs 0	RRMS new lesions 4% vs 1% PPMS 0 vs 0
**Rituximab**					
COMBAT-MS and Multiple MS [58]	Single-center prospective observational study with 4.2 year follow-up	RRMS < 8 months–90 8–12–144 12–18–242 > 8 months–160	< 8 months 8–12 months 12–18 months > 18 months	Relapse rate 0.9%, 0.3%, 0.3% 0.8%, respectively	NET2L 27.8%, 4.8%,1.7%,5%, respectively

*EID* extended interval dosing, *SID* standard interval dosing, *NET2L* new and enlarging T2 lesion; *BPV* brain parenchymal volume; *GdE* gadolinium-enhancing lesions; *ARR* annualized relapse rate; *CWD* confirmed worsening of disability; *CDI* confirmed disability improvement; *HR* hazard ratio; *OR* odds ratio; *MDI* median interval; *vs* versus

## Reducing dosing

Relatively limited data exist on reduced dosing of DMT during treatment of pwMS. In a small case series, changing to every other or every 3 day fingolimod dosing, due to increased LFTs or decreased lymphocyte count to ≤ 0.2 × 10^9^/L, resulted in reversal of laboratory abnormalities. Although none of eight patients on lower dose developed clinical relapses, five of eight patients had new MRI lesions [59]. A retrospective study of 36 pwRRMS taking fingolimod nondaily for at least 6 months because of low lymphocyte count demonstrated an increase in the mean lymphocyte count without significant differences in clinical disease activity versus 36 propensity score-matched patients on daily dose [60]. However, fingolimod, 0.5 mg, demonstrated superior clinical efficacy compared with fingolimod 0.25 mg and glatiramer acetate, 20 mg (ARR 0.15 vs 0.22 vs 0.26) in a 12-month study of two fingolimod doses vs glatiramer acetate for the treatment of pwRRMS [61].

Similarly, 50% dose reduction of dimethyl fumarate (120 mg twice/day) for 4 weeks due to poor tolerability was not clinically effective. Nonetheless, temporarily reducing the DMF dose may increase the likelihood that patients can remain on therapy [62]. A report of 30 pwMS with IgG hypogammaglobulinemia during anti-CD20 treatment with dose reductions from 25% to 50% of standard doses (500–750 mg for rituximab and 300 mg for ocrelizumab) did not result in IgG increase [63]. However, the dose reduction group experienced fewer infections. A prospective single-site 12-month reduced dosing study of 59 MS patients (37RRMS, 22SPMS) who had been using rituximab 1000 mg/infusion every 6 months and then received a 500 mg/infusion in the subsequent 12 months reported no relapses, changes in EDSS, or changes in serum NfL levels in the 12 months of 50% reduced rituximab dosing [64]. In this study, IgG concentrations were significantly lower in those with the highest life-time exposure to rituximab. IgG-deficient patients had the greatest risk of infections (OR 6.27, 95% CI 1.71–22.9, *p* = 0.005), supporting the concept of reduced dosing.

## Discontinuation studies in MS

Shifts in the risk-to-benefit ratio for MS DMTs with increasing age have led to the discussions of discontinuation of DMTs, and have prompted studies to test the effect of DMT discontinuation in older pwMS [65] (Table 4). An investigation of DMT discontinuation in 221 pwRRMS found that those with age ≥45 years, DMT intake ≥4 years, and without evidence of clinical or radiological disease activity were the most likely to remain relapse-free after DMT discontinuation [66]. A retrospective, observational study of 178 pwMS aged over 60 who discontinued MS DMTs reported only one relapse within variable follow-up times, but MRI data were not usually available within 2 years of discontinuation. Notably, 10.7% of those who stopped DMT reinitiated DMT later [67]. Similarly, studies of discontinuation of DMTs including  SPMS > 50  years did not find differences in time to first relapse and disability progression between continuers and discontinuers [68, 69]. A study of 69 pwRRMS who discontinued DMTs (typically first generation DMTs) compared their outcomes to those of 69 patients who remained on DMT, and were otherwise matched by age, sex, treatment, treatment duration, disease duration, and EDSS score. Univariate and multivariable Cox proportional hazard models found a significant difference in the association between young (< 45 years) and old (> 45 years) patients for time to new MRI or clinical activity. Discontinuation after age 45 was associated with higher rate of a stable disease course [70]. However, a study of 216 MS patients from New York State Multiple Sclerosis Consortium found that 32.9% of pwMS who discontinued DMT experienced disability worsening or progression. The rate of disability worsening was only slightly higher in patients younger than 55 years versus those older than 55 years who discontinued DMT (31.1% vs 25.9%, respectively). MS patients with EDSS ≥ 6.0 had greater disability worsening when compared to less-disabled patients while remaining on therapy as well as after discontinuation [71]. Our own earlier study on relapse and disability worsening after discontinuation of interferon-beta showed that 65% of patients experienced a relapse within 34 months after stopping interferon [72]. The analysis of patients who discontinued DMT (73% interferons) from MSBase registry found that risk of post-DMT relapse was higher in younger female patients, those with moderate disability, and a relapse within 1 year prior to discontinuation [73]. Chappuis et al. [74] investigated the rate of relapse within 1 year after discontinuation of first- and second-line drugs in middle-aged patients (median 52.8 years). The probability of having a relapse was 6% for the first-line DMT discontinuation, 9% for fingolimod and 43% for natalizumab, highlighting the risk of “rebound” inflammatory activity after discontinuation of natalizumab. DOT-MS and DISCOMS were the only published randomized, controlled studies investigating the consequences of DMT discontinuation in an older MS population [75, 76]. The mean age of DOT-MS population was 54 years versus 63 in DISCOM. Both studies recruited patients with no relapse within the past 5 years and no new MRI lesion in the past 3 or 5 years. DOT-MS trial was discontinued because of return of inflammatory activity after treatment stopping [75]. DISCOMS recruited 259 patients with mean age of 63 years. Six (4.7%) of 128 participants in the group randomized to continue DMT and 16 (12.2%) of 131 in the discontinue group had a relapse or a new or expanding brain MRI lesion within the 2-year study. No difference was observed between groups in disability progression (11.1% vs 12.3%, respectively), with similar rates of adverse events (85% vs 79%). The authors concluded that discontinuation of DMTs might be an option in patients older than 55 years who have stable MS [76]. DISCOMS was relatively small, short in duration, and lacked systematic use of contrast in all study MRIs. The differences in outcomes between DOT-MS and DISCOMS might be related to younger participants in DOT-MS and inclusion of patients with recent “non-significant” MRI lesions in this study. A recent observational cohort study of 154 propensity score-matched pwMS from the French MS registry, with age > 50 years and lack of inflammatory activity for 4.4 years (much shorter in comparison with DOT-MS and DISOMS), who continued or stopped high-efficacy drugs with potential of rebound after discontinuation, contrary to DOT-MS and DISCOMS with no or low rate of high-efficacy drugs, showed that time to first relapse within 2 years was significantly reduced for those discontinuing natalizumab (HR = 7.2) and fingolimod (HR = 4.5) but not for anti-CD20 (HR = 1.1) [77]. These results might reflect observation that younger age is an important factor for recurrence or rebound of MS activity after natalizumab and fingolimod discontinuation [78]. A recent meta-analysis of 22 studies assessing the rate of disease reactivation and associated risk factors after discontinuation of DMTs concluded that the risk for relapse is < 1% in pwMS after age 60, with > 10 years of DMT use or 8 years of disease stability [79].

**Table 4:** Studies of age-dependent effects on DMT discontinuation in MS

Study authors *N* MS subtype Mean age	Type of study	Length of DMT treatment; DMTs used before discontinuation	Length of observation after discontinuation	Outcomes in relation to age
Bsteh et al. [66] *N* = 221 RRMS Mean age 37.9	Retrospective study of prospectively collected data	> 12 months; Most on IFNb and GA	≥ 2 years after discontinuation, mean 4.2 y	After median follow-up 3.8 years, 98 patients (44.3%) had relapse but only 14/56 (25%) aged ≥ 45 y relapsed after discontinuation Age > 45 years plus no relapses in prior ≥ 4 years on DMT had very low HR=0.06 prediction for relapse, (p < 0.001) and low for disability progression (HR=0.65)
Birnbaum G [65] *N* = 94 SPMS, RRMS Mean age 61 y SPMS; 49 RRMS	Prospective observation	11 years SP 7 years RR	4 years SP 2.5 years RR	Active disease (relapse + MRI) 11,7% SPMS and 58.8% RRMS Stable disease > 61 years
Bonenfant et al. [69] *N* = 100 SPMS Mean age 47.2	Retrospective one arm (IFNb, GA)	60.4 months	62.4 months	Low ARR 0.07 Age > 50 years decreased risk for active disease HR = 0.8
Hua et al [67] *N* = 178 MS ≥ 60 yo (mean 68.1)	Retrospective study from 3 centers for 2010–2016	> 2 years; > 90% on IFNb or GA at some point	2 years	> 60 y; relapse rate 0.6% (only one relapse); 10.7% reinitiated DMT; 95.1%had no NET2L 85.1% patients had no new GdE lesions post-discontinuation
Yano et al. [70] *N* = 69 (69 matched controls); > 18 y; Mean age 44.6y	Retrospective study of prospectively collected data; case-controlled	> 2 years; most on IFNb or GA	4 years	> 45 y associated with better disease course after discontinuation. Shorter time to MRI event (HR = 1.64) and time to disease activity (HR = 1,84) with age <45 y
Kaminsky et al. [68] *N* = 498 (132 vs 366) > 50 years	Retrospective study with propensity scoring (IFNb, GA, Fingo, DMF, TERF)	4.5 years	7.7 years	No difference in time to first relapse between dis- and continuers ARR 0.21 vs 0.23 EDSS increase in 45.1% vs 45.5% patient (HR=0.89)
NYSMSC [71] *N* = 216 RRMS, SPMS Mean age 50.6	Retrospective study of longitudinal cohort	Mean 32.2 months; Most on IFNb or GA	Mean 24.2 month	> 55 vs < 55 y; CDW 31.1% vs 25.9%
MSBASE [73] *n* = 4842 Age > 18; Median age 35.9y	Retrospective study of prospectively collected data from 20 centers	> 12 months; All available DMTs, most on IFNb or GA	> 24 months; median 5.6y	For each additional year older at baseline 3% reduction in relapse rate after DMT discontinuation
Chappuis et al [74] *N* = 232 All MS subtypes > 45 y Median age 52.8 y	Retrospective study of prospectively collected data	> 6 months; All available DMTs	> 12 months; Median 6.4 y after first-line DMT and 4.2 y after second-line DMT discontinuation	Relapse HR: 45–50 years HR 1 50–55 years HR 0.80 > 55 years HR 0.66 shorter time to MR activity before 55 years than > 55 y
DISCOMS [76] *N* = 259 Any MS subtype (83% RRMS); Median age 63 y	Randomized, single blinded prospective, multi-center; age > 55 y	5 years; most on IFNb or GA	> 18mon: assigned 1:1 to discontinue or continue DMT	Continue vs discontinue groups: Relapse/NET2L: 4∙7% vs 12∙2%; CDW 11.1 v. 12.3%
Jouvenot et al. [77] , *N* = 154 discontinuing, *N* = 154 continuing after propensity score match RRMS, Mean age 57.7y	Observational cohort 38 study centers. Propensity matched design, age > 50 y	5.6 years HET (NTZ, Fingo, Rtx, OCR)	2.5 years	HR for time to first relapse: NTZ = 7.2, Fingo = 4.5 and anti-CD20 = 1.1

*IFNb* beta interferon; *GA glatiramer*, *TERF* teriflunomide; *DMF* dimethyl fumarate; *Fingo* fingolimod; *NTZ* natalizumab; *OCR ocrelizumab, RTX* rituximab; *NET2L* new and enlarging T2 lesion; *HR* hazard ratio; *CDW* confirmed disability worsening; *ARR* annual relapse rate

Guidance for when DMTs might safely be stopped was addressed using prospectively collected data in relapsing MS patients who received interferon-beta or glatiramer acetate. Three factors—age (especially over 55), MRI stability for at least 4 years, and clinical stability—predicted no disease reactivation [80]. Another study demonstrated that pwMS that show an increase in the serum levels of neurofilament-light chain and glial fibrillary acidic protein after DMT cessation were more likely to experience clinical or radiological disease reactivation [81].

However, the sensitivity of NfL elevation may not be sufficient for this purpose. Recent data on correlation between enhancing MRI lesion and NfL levels during the 24-week natalizumab treatment interruption period in RESTORE trail showed that most participants with Gd + lesions did not have elevations in sNfL levels [82]. Thus, at this time, serum biomarkers are of uncertain value in predicting risk of disease recurrence after stopping DMT.

## Conclusions and recommendations

We believe that it is time to re-address the need for continued DMT in older pwMS. Accumulating knowledge on the impact of aging on the immune system is in accord with the observed diminished MS activity with age. At the same time, in older pwMS, a number of unfavorable processes negatively influence treatment risks. The increased vulnerability to infections with aging intensifies the increased risk for infections seen with some DMTs. Although many de-escalation strategies now exist such as extended dosing, switching from high-efficacy drugs to less-effective DMTs with lower risks, reduced dosing, and, in some cases, DMT discontinuation, none are definitive. A definite conclusion about DMT discontinuation in older pwMS awaits future multi-year prospective randomized-controlled trials with more patients studied for longer durations. The characteristic of drug being discontinued should also be considered. Three studies are underway now, long-term continuation in DISCOMS extension, STOP-I-MS in SPMS patients, and OCR-discontinuation. Until we have better data, the decision to discontinue DMTs will require active involvement of the patient, discussion of all potential safety issues, and weighing benefits against risks for each individual decision. Close monitoring of MS patients following DMT discontinuation is recommended, including neurological assessments every 6 months, and a surveillance MRI approximately 6 months after discontinuation, followed by yearly surveillance MRIs for at least 3 years. A de-escalation strategy would likely have positive pharmaco-economic implications, since it should reduce healthcare costs.

Ideally, comparison of de-escalating versus continuation versus discontinuation of DMTs should be done by large prospective randomized-controlled trials of sufficient duration and with sufficient patient numbers to allow comparisons at different ages beyond 50 or 55 years. Such studies should enroll enough pwMS to stratify results based on sex, race, comorbidities, MRI burden, biomarker results, and other factors. These studies should also aim to identify the best monitoring strategy following DMT discontinuation. Because people age differently, incorporation of testing for specific markers of immunosenescence (such as T-cell receptor excision circles or leukocyte telomere length) may allow further stratification of results, and better ability to apply study results to individual patients and potentially discriminate between chronologic and biologic age [27].
